# Editorial Note: A dual-channel secondary closed-loop supply chain considering retail groups and fairness concerns

**DOI:** 10.1371/journal.pone.0329845

**Published:** 2025-08-07

**Authors:** The PLOS One Editors

Following the publication of this article [1], it came to light that the article’s pre-publication peer review did not meet the expected standards for *PLOS One*. We regret that the issues were not identified and addressed prior to the article’s publication.

This note is not intended to question the quality of the article or contributions of the authors to the review process, as the authors had no involvement in the peer review.
